# R-loop-forming Sequences Analysis in Thousands of Viral Genomes Identify A New Common Element in Herpesviruses

**DOI:** 10.1038/s41598-020-63101-9

**Published:** 2020-04-14

**Authors:** Thidathip Wongsurawat, Arundhati Gupta, Piroon Jenjaroenpun, Shana Owens, J. Craig Forrest, Intawat Nookaew

**Affiliations:** 1 Department of Biomedical Informatics, College of Medicine, University of Arkansas for Medical Sciences, Little Rock, Arkansas USA; 2 Department of Microbiology and Immunology and Center for Microbial Pathogenesis and Host Inflammatory Responses, University of Arkansas for Medical Sciences, Little Rock, Arkansas USA; 3 Present Address: Department of Pediatrics, University of Pittsburgh School of Medicine, Pittsburgh, PA USA

**Keywords:** Computational biology and bioinformatics, Genomics

## Abstract

R-loops are RNA-DNA hybrid sequences that are emerging players in various biological processes, occurring in both prokaryotic and eukaryotic cells. In viruses, R-loop investigation is limited and functional importance is poorly understood. Here, we performed a computational approach to investigate prevalence, distribution, and location of R-loop forming sequences (RLFS) across more than 6000 viral genomes. A total of 14637 RLFS loci were identified in 1586 viral genomes. Over 70% of RLFS-positive genomes are dsDNA viruses. In the order *Herpesvirales*, RLFS were presented in all members whereas no RLFS was predicted in the order *Ligamenvirales*. Analysis of RLFS density in all RLFS-positive genomes revealed unusually high RLFS densities in herpesvirus genomes, with RLFS densities particularly enriched within repeat regions such as the terminal repeats (TRs). RLFS in TRs are positionally conserved between herpesviruses. Validating the computationally-identified RLFS, R-loop formation was experimentally confirmed in the TR and viral Bcl-2 promoter of Kaposi sarcoma-associated herpesvirus (KSHV). These predictions and validations support future analysis of RLFS in regulating the replication, transcription, and genome maintenance of herpesviruses.

## Introduction

Recent advances in the field of genomics have revealed widespread occurrence of non-canonical nucleic acid-forming structures, such as Z-DNA^1^, hairpin loops^2,3^, and G-quadruplexes (guanine-rich sequences that attain specific four-stranded conformations)^4^, in various genomes including viruses^5^. These structures likely play important roles in the replication strategies used by a particular virus. For instance, Z-DNA structures in the simian virus 40 enhancer regions activate transcription^1^. Hairpin structures at the termini of the adeno-associated virus genome promote persistent DNA circles and concatemers during recombination processes that occur in the infected host cell^2,3^. G-quadruplexes are present in several viral genomes e.g., human immunodeficiency virus (HIV- 1), Epstein–Barr virus (EBV), and human papillomavirus (HPV) (reviewed in^4^ and function in various aspects of the replication cycles of these viruses.

Another non-canonical nucleic acid structure called an R-loop or RNA:DNA structure is preferentially formed within G-rich sequences and possesses greater thermodynamic stability than the original DNA:DNA duplex^6^. R-loops have been experimentally observed in a wide range of organisms, from bacteria to mammals^6,7^, where they function in transcription^8^, telomere maintenance^9^, genome instability^10,11^, and epigenetic regulation^12^. They are also associated with certain diseases, such as Prader–Willi syndrome^13^, ataxia with oculomotor apraxia^14^, amyotrophic lateral sclerosis^15^, spinal muscular atrophy^16,17^, motor neuron disorders^18^, cancers^19^ and many others^20^. R-loops, have been predicted by computational approaches in various organisms^21–23^ and demonstrated by direct experimental evidence, however, genome-scale identification of R-loops in the viral species has not been explored.

Knowledge regarding roles for R-loops in viruses is limited. In 2011, Rennekamp *et.al*. demonstrated the persistence of R-loops at the origin of replication of Epstein–Barr virus (EBV)^24^. Removal of R-loops by RNase H, an RNA-degrading enzyme specific for RNA-DNA duplex molecules, eliminated the generation of ssDNA at the viral origin, thereby inhibiting viral replication by preventing the recruitment of the ssDNA binding protein BALF2 to the origin of viral replication^24^. In 2014, Jackson *et al*. demonstrated that R-loops formed during infection by Kaposi sarcoma-associated herpesvirus (KSHV) correlated with a host-cell DNA damage response and genome instability^25^. The mechanism for this likely involves KSHV ORF57, a protein that hijacks the host hTREX complex, an RNA binding protein complex that normally prevents R-loop formation, leading to enhanced R-loop formation and DNA damage in KSHV-infected cells. Although, this study did not report R-loop forming loci in the KSHV genome, it provides important evidence that R-loop formation in virus-infected cells might be impacted by the virus life cycle. Notably, both studies highlighted the investigation of R-loop formation in herpesviruses.

In this study, we employed our previously developed computer algorithm, QmRLFS-finder^26^, to identify possible R-loop forming sequences (RLFSs) in a reference set of over 6000 viral genomes. The genome set was retrieved from the National Center for Biotechnology Information (NCBI) database and exhibits remarkable diversity in length and information content^27^ across a wide range of viral families. We found that approximately 25% of the total dataset contains at least one RLFS in a specific genome with an expected overrepresentation in dsDNA viruses compared to other virus types, especially in the *Herpesviridae* order. The distribution and association of RLFS in herpesvirus genomes and unique genomic features including coding, non-coding regions, and repeat regions were further investigated and experimentally validated to highlight potential roles for R-loops in the herpesvirus life cycle.

## Materials and Methods

### Data acquisition and data availability

We used a set of viral genomes available on 17^th^ Jan, 2017 from NCBI (http://www.ncbi.nlm.nih.gov). We retrieved the DNA sequences (for RNA viruses, NCBI provide DNA sequences version of the RNA genome) of the genomes from the database and preprocessed following Zhang *et al*.^27^, obtaining a total of 6153 viruses that will be used in further steps. Viral taxonomic classification data (i.e., The Baltimore classification and the International Committee on Taxonomy of Viruses or ICTV classification) was also collected from NCBI taxonomy database (http://www.ncbi.nlm.nih.gov/taxonomy)^28^ and used for further analysis and interpretations.

### R-loop forming sequence (RLFS) prediction by QmRLFS-finder

We predicted possible R-loop forming structures on both strands of genomic DNA/RNA using our QmRLFS-finder software^26^. Briefly, QmRLFS-finder identify RLFS based on three structural features in the sequence beginning with a short G-cluster sequence is responsible for initiating R-loop formation (R-loop initiation zone), then a linker sequences that connect to a downstream region of G-rich sequence as called R-loop elongation. The predicted RLFSs that are overlapped at least one nucleotide were then merged together to be one RLFS. The number of RLFSs found in each genome was reported and available in Supplementary Table 3. The RLFSs located in the coding sequence (CDS) and non-CDS are identified using BEDtools2^29^. Visualization of the predicted RLFS results and statistical analysis were performed under R suite software (https://www.r-project.org/) and Bio-Graphics module (https://metacpan.org/release/Bio-Graphics) in perl language.

### Cell culture and virus

The iSLK and iSLK-BAC16 cells, which are derived from human endothelial cells, were a gift from Dr. Jae Jung^30^. The cells were cultured in Dulbecco’s Modified Eagle’s Medium supplemented with 10% FBS, 1% L-Gln, 100U/ml of penicillin, 100U/ml of streptomycin, 250μg/ml of geneticin, and 1μg/ml of puromycin. 1200μg/ml hygromycin B was added to iSLKs-BAC16 culture media to maintain KSHV. Cells were cultured at 37 °C with 5% CO_2_ and ~99% humidity. KSHV lytic replication was induced by replacing culture media with DMEM supplemented with 10% FBS, 100 U/ml penicillin, 100 μg/ml streptomycin, 2 mM L-glutamine, 1 μg/ml doxycycline and 1 mM sodium butyrate. Cells were induced for 48 hours. Induction of the lytic cycle was confirmed by quantitative PCR for the KSHV genome, which revealed a >30-fold increase in viral genomes relative to cellular genomes by 48 h post-induction.

### Extraction of nuclear materials

A total of 10^7^ iSLK or iSLK-BAC16 cells were lysed in 500μl lysis buffer (0.5% SDS, 50 mM NaCl, pH 8.0) with 1 unit of proteinase K (New England Biolabs or NEB) at 37 °C overnight. Total nucleic acids were precipitated with 1.5 M NaCl and isopropanol, and resuspended in 500μl TE buffer. 50μg of genomic DNA was digested using a mixture containing 10 units each of *XbaI*, *XhoI*, *EcoRI* and *BamHI* (NEB) in CutSmart Buffer (NEB) at 37 °C overnight. Each sample was split into two, and one half was digested with RNase H (NEB) at 37 °C for 8 hours. Nucleic acids were purified from all samples by phenol:chloroform extraction followed by ethanol precipitation. Purified material was resuspended in IP buffer (50 mM Tris-Cl, 150 mM NaCl, 1% NP-40, pH 8.0).

### DNA-RNA Immunoprecipitation (DRIP)

DRIP was performed essentially as previously described with minor modifications^31^. In brief, 10μg of DNA was incubated with 4μg of S9.6 (Kerafast) or isotype control (mouse IgG) in 500μl IP buffer at 4 °C overnight. 30μl of Dynabeads Protein G beads (Thermo Fisher Scientific) was added to each sample, and samples were rotated for 1 h at room temperature. Antibody-DNA-RNA complexes were pelleted and were eluted in 200μl of elution buffer provided in the kit. Precipitation complexes were digested for 2 h with proteinase K, and nucleic acids were purified by phenol:chloroform extraction followed by ethanol precipitation. Recovered fragments were analyzed by PCR and qPCR.

### Drip-PCR

Samples were analyzed for the presence of two predicted RLFS in the KSHV genome at the *ORF16* promoter and the terminal repeat (TR). A previously defined *c-MYC* RLFS was used as a positive control to ensure successful DRIP^19^. Primer pairs were designed with Primer-BLAST^32^. A 206nt segment within ORF45 (nt 67600 to 67805 in NC009333.1) served as a negative control for R-loops, as there are no predicted RLFS at this locus. PCR was performed using Hot Start 2x Taq mastermix (NEB) on an Applied Biosystems SimpliAmp thermal cycler (Thermo Fisher Scientific) using the following reaction parameters: 5 min at 95 °C followed by 30 cycles of 15 s at 95 °C, 15 s at 60 °C, and 30 s at 72 °C. PCR products were resolved by electrophoresis on 1.2% agarose. Primer pairs were as follows. *ORF16*-promoter: forward 5′-ACATTTGCCCCACCGTCGCCT-3′; reverse 5′- GCACATAGCACGCGCACAGCA-3′. Terminal repeat: forward 5′-GACCCCGGGCAGCGAGGGAA-3′; reverse 5′-AGGGCTCCACGTAGCAAGCACTG-3′. ORF45 (absence of predicted RLFS): forward 5′-GGGAGGTGACCCTTTGTGCT-3′; reverse 5′-CTCATGGACGTGGGCCAGA-3′.

### qPCR

Genome copy number was determined by qPCR relative to uninduced iSLK-BAC16 samples to verify induction of the KSHV lytic cycle in iSLK-BAC16 cells treated with doxycycline and sodium butyrate. Quantitative PCR was performed with RT^2^ SYBR Green ROX qPCR mastermix (Qiagen) on an Applied Biosystems QuantStudio 6 Flex thermal cycler (Thermo Fisher Scientific) using the following reaction parameters: 10 min at 95 °C followed by 40 cycles of 15 s at 95 °C and 1 min at 60 °C. Data were analyzed using the percentage of input method where ΔCt = Ct_input_- Ct_IP._ Results are expressed as 100*(2^ΔCt^).

## Results

### R-loop forming sequences are predominant in dsDNA viruses

**Figure 1:**
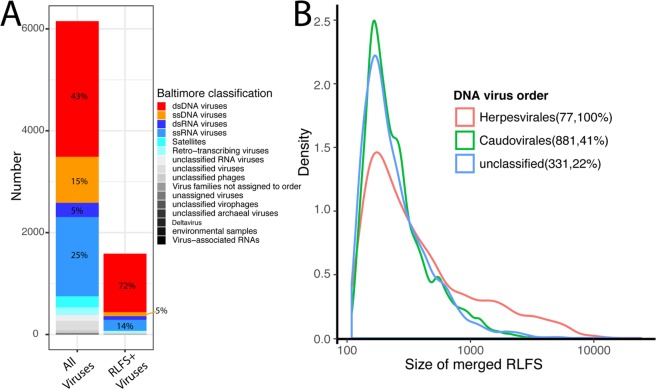
Distribution RLFS-positive genome in viral kingdom. (**A**) Bar chart shows the distribution of all viral genomes in the dataset with the RLFS-positive genomes. The colors represent the different group of viruses based on Baltimore classification. (**B**) Density plots show the distribution of size of detected merged RLFS in different order level of DNA virus based on ICTV classification. The numbers and percentage values in parenthesis represent the number of RLFS-positive genome and percentage RLFS-positive genome of for each order, respectively.

**Figure 2:**
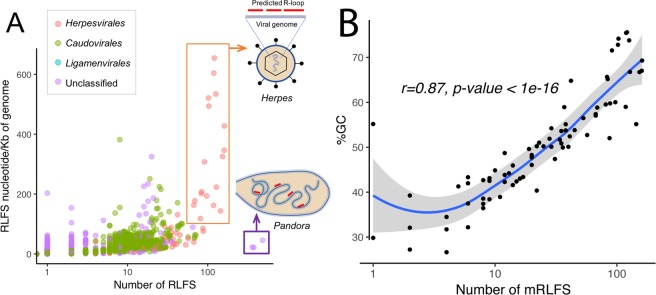
Association between indentified mRLFS number with mRLFS density on the genome or %GC content. (**A**) Scatter plot shows mRLFS density (mRLFS nucleotide per kilobase of genome) versus number of mRLFS of individual RLFS-positive genome. Red and magenta box indicate herpesviruses and pandoraviruses, respectively that illustrated in cartoon picture. Blue and red bars represent genome and mRLFS (**B**) Scatter plot shows strong correlation between %GC content and number of mRLFS of all 77 herpesvirus genomes in the reference dataset.

We analyzed a total of 6153 viral genomes, which are 2663 double-stranded DNA (dsDNA), 1556 single-stranded RNA (ssRNA), 905 single-stranded DNA (ssDNA), 278 double-stranded RNA (dsRNA), and 751 other viruses based on the Baltimore classification scheme (Supplementary Table 1). We predicted RLFSs and merged them if they overlapped to each other (at least one nucleotide). All RLFSs which passed merging process were called merged RLFS (mRLFS). From the dataset, we identified 14637 mRLFS found in 1586 genomes. From these genomes, 1148 genomes were dsDNA (72% of total) followed by 216 (14%), 78 (5%), and 71 (4%) in ssRNA, ssDNA, and dsRNA, respectively (Fig. 1A). Even though R-loop formation in the genome of ssRNA and dsRNA viruses is not logically possible, the predicted results may be useful for study of RNA viruses that have RNA-DNA hybrid structures during their replication (e.g. human immunodeficiency virus type 1)^33^. We then focused on the genomes of DNA viruses for further analysis. In the reference dataset, there are 3 orders of DNA virus with unclassified order (see Supplementary Table 2); surprisingly, *Ligamenvirales*, which are linear dsDNA genome viruses known as an archaea infectious agent, had no RLFS-positive genome. In contrast, all viruses in the *herpesvirales* order, which consists of eukaryotic dsDNA viruses found only in animal hosts, contain predicted RLFS within their genomes, and the size of mRLFS in these viruses was longer than others viral families (Fig. 1B). Very long mRLFS were found in the highly G-rich repetitive regions of herpesviruses (see Fig. 2). The details of the number of RLFS-positive and RLFS-negative genomes in each family are provided in Supplementary Table 3.

### Herpesvirus genomes have unusually high RLFS density

A key factor influencing detection of RLFS in any sequence is genome length, which increases the probability of finding RLFS over G-rich regions^6,7^. We therefore investigated the association between number of detected mRLFS relative to viral genome size and GC content for the RLFS-positive genomes of DNA viruses. Across all DNA viral genomes, the giant viruses, *Pandoravirus dulcis*, *P. salinus*, and *P. inopinatum* contained the largest number of mRLFS: 482, 375, and 358 mRLFS, respectively (Supplementary Table 4). On the other hand, only one mRLFS was detected in several of very small genomes such as virus satellites. The mRLFS density (mRLFS/kb of sequence) in a given viral genome is plotted as a function of total numbers of mRLFS per virus in Fig. 2A. While pandoraviruses have the largest number of mRLFS in their genomes, this corresponds to only 9% of their genome length. However, herpesviruses have both high numbers of mRLFS and high coverage of the genome (Fig. 2A). Consistent with the observation that R-loops are highly associated with G-rich sequences^6,7^, we found a strong correlation (r = 0.87, p-value <1e-16) between the number of mRLFS and the GC content of herpesvirus genomes as shown in Fig. 2B. We then compared the identified mRLFS in herpesviruses with randomly shuffled DNA sequences (n = 30) from each individual herpesvirus genome that preserved GC content and genome length illustrated in Supplementary Fig. 1. We found that the number of mRLFS identified in herpesviruses was higher than in the random sequences when GC content was below 65%. On the contrary, when GC content was greater than 65%, fewer mRLFS were identified in the herpesviruses than in the randomized sequences. This indicated an evolutionary basis for the existence of non-random GC clusters pattern in herpesvirus genomes.

### mRLFS are common in terminal repeat regions of herpesviruses

*Herpesviridae* is composed of three subfamilies (*Alphaherpesvirinae*, *Betaherpesvirinae*, and *Gammaherpesvirinae*), whose genome sizes range from 120 to 230 kbp and contain approximately 60 to 120 ORFs. We investigated the distribution of mRLFS for individual genomes, including coding sequence (CDS), non-CDS, and repeat regions of all *herpesviridae* members (Supplementary Fig. 2). We found a strong positive correlation (r = 0.88) between GC content and the number of mRLFS found in CDS. In contrast, non-CDS exhibited a negative correlation (r = −0.57) between GC content and the number of mRLFS. However, the distribution pattern of mRLFS in CDS and non-CDS was variable between different herpesviruses (Supplementary Fig. 2). It is thus difficult to comment on the possible functions of Rloop in CDS vs non-CDS.

Of note, our computational-based approach predicted the presence of an RLFS in the Epstein–Barr virus (EBV, also known as human gammaherpesvirus-4) origin of replication (Supplementary Fig. 3). This R-loop was previously reported and demonstrated to facilitate viral DNA replication by enabling binding of BALF2 protein to the origin of replication^24^. This observation provides an independent experimental validation of our prediction tool and provides confidence on the identified RLFS by the computational approach presented here.

**Figure 3:**
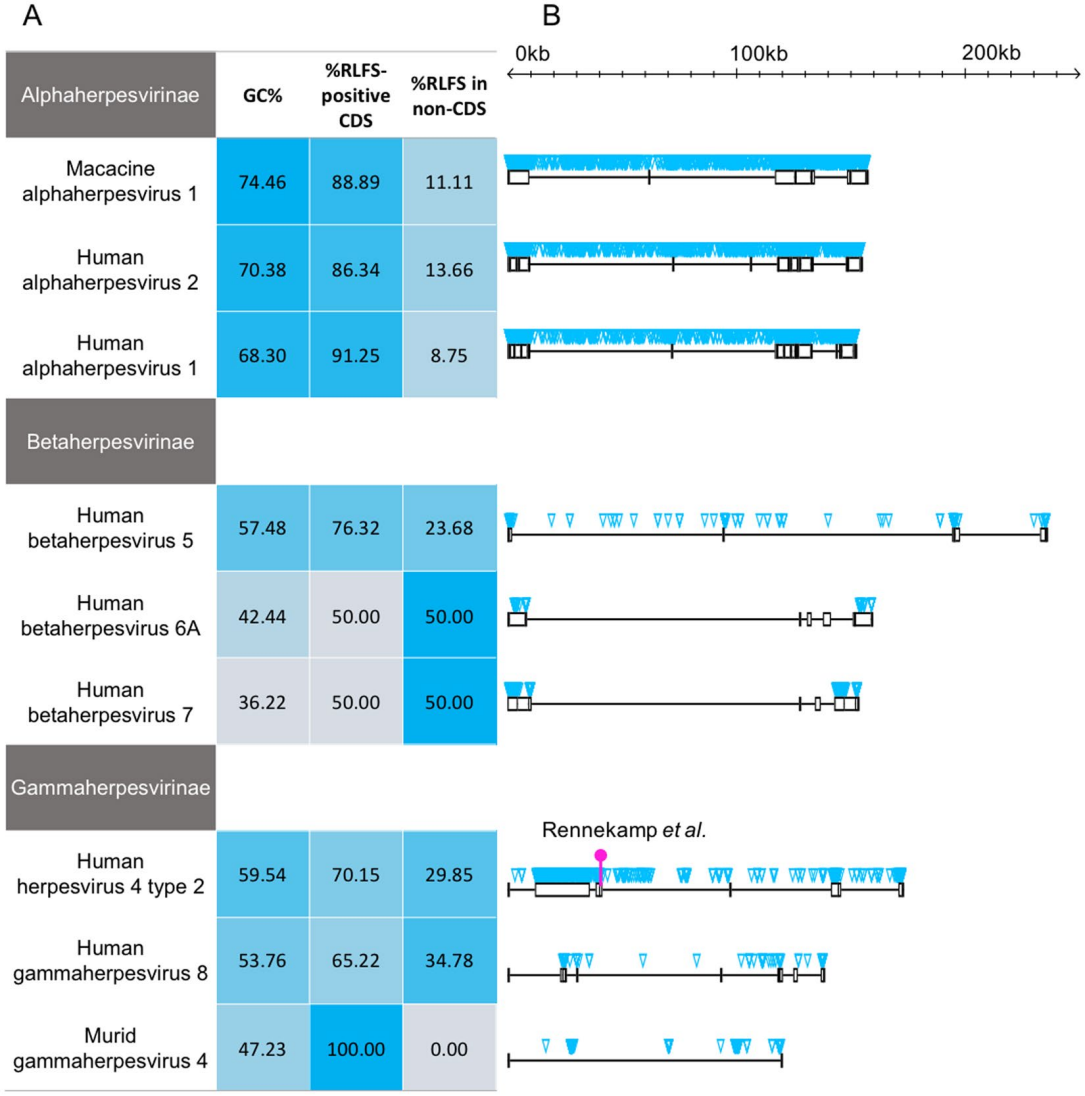
Schematic representation of the distribution of mRLFS in selected representative herpesviruses in each subfamily. (**A**) Heat map plots. From the left to the right column represent percent of GC content, percent of mRLFS found in CDS and percent of mRLFS found in non-CDS, respectively. (**B**) Distribution of mRLFS in the genome. The genomes were drawn to scale. The black line represented the genomes with open boxes represent the repeat regions in the genome. The blue triangles represent the genomic positions of mRLFS. The magenta mark is the previously known R-loop forming locus on the Epstein–Barr virus (EBV) genome^24^.

Herpesvirus genomes contain repetitive regions, especially at the termini of the linear genomes (terminal repeats, TR) that play crucial regulatory roles in genome maintenance^34^. Mapping mRLFS on repetitive regions of representative genomes from each subfamily (Fig. 3) revealed a high degree of overlap between mRLFS and the 3′ TR that were conserved across the different subfamilies (see Supplementary Fig. 4 for all 77 genomes). The sequence patterns of mRLFS predicted in the 3′ TRs of herpesvirus genomes resemble those of experimentally determined mRLFS found in mammalian immunoglobulin switch regions, which is also an R-loop prone region (Supplementary Fig. 5)^35^. In addition, some herpesvirus genomes have TTAGGG repeats identical to host cell telomere repeats^36,37^. R-loops have also been detected in the telomere repeat elements of host organisms and have an essential role in telomere maintenance^34^. Similar to host genomes, herpesvirus TRs are essential for viral genome stability^38^. This raises the question whether herpesvirus R-loops serve the same function as host R-loops.

### Experimental validation of select RLFS loci in the KSHV genome

**Figure 4:**
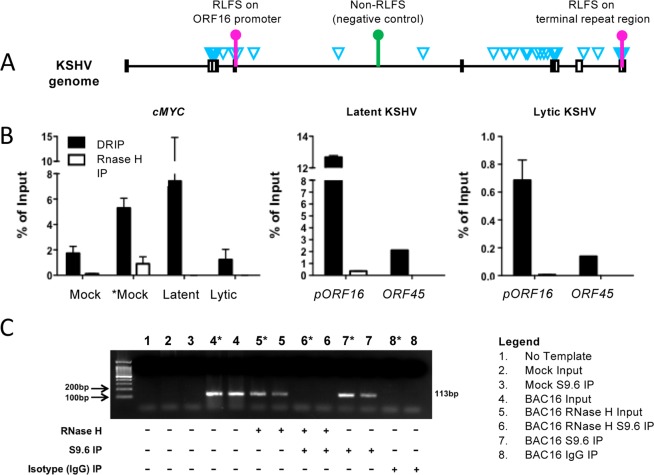
R-loop formation in Kaposi sarcoma-associated herpesvirus (KSHV) genome. Total nucleic acid from iSLK cells latently infected with KSHV, undergoing lytic viral replication, and uninfected cells (mock) were subjected DNA-RNA immunoprecipitation with S9.6 antibody. RNase H digestion was used as a negative control to ensure S9.6 was specific for DNA-RNA hybrids. A mouse IgG served as an isotype control to ensure DNA-RNA binding to S9.6 was specific. (**A**) Schematic representation of KSHV genome. Selected loci of RLFS and non-RLFS for experimental validation are shown in pink and green mark. The blue triangles represent the positions of RLFS. The boxes represent the repeat regions in the genome. (**B**) qPCR was performed to measure specific immunoprecipitation of KSHV *ORF16* promoter by S9.6, *cMYC* was used as a positive control, and a region of KSHV *ORF45* was used as a negative control. RNase H digestion was used as a treatment control to ensure S9.6 was specific for DNA:RNA hybrids. (**C**) R-loops were detected in the terminal repeat region of KSHV genomes by PCR. *indicates lytic BAC16. Reactivated (lytic) samples were treated with sodium butyrate and doxycycline to induce KSHV lytic replication 48 h prior to cell lysis. Data are presented as mean of technical duplicates. Error bars represent SD. Experiments were performed in biological duplicates, only one replicate shown.

To experimentally validate the newly predicted mRLFS in herpesvirus genomes, we evaluated select genomic regions of the Kaposi sarcoma-associated herpesvirus (KSHV, also known as human gammaherpesvirus-8). Two predicted RLFS-positive regions, the promoter for *ORF16* (viral Bcl2) and within the TR, were selected for validation based on their published importance in KSHV infection (Fig. 4A). A third region that corresponded to *ORF45* (nucleotide 67600 to 67805 in the NC009333.1 KSHV genome sequence) was not predicted to encode RLFS and was chosen as a negative control. We performed DNA:RNA immunoprecipitation (DRIP) using antibody S9.6, which specifically recognizes DNA:RNA hybrid molecules, on (i) uninfected iSLK cells, (ii) induced uninfected iSLK cells (iii) iSLK-BAC16 cells latently infected with KSHV and (iv) induced iSLK-BAC16 cells undergoing lytic viral replication. As specificity controls, parallel DRIP was performed on isolated nucleic acids treated with RNase H to degrade DNA:RNA hybrids. A previously identified RLFS in the cellular *c-MYC* promoter was evaluated as a positive control for DRIP. For *ORF16* and *c-MYC*, quantitative PCR (qPCR) demonstrated superior precipitation in samples not treated with RNase H in comparison to RNase H digested samples, while the negative control *ORF45* sequence was only minimally detected in S9.6 precipitates (Fig. 4B). These data therefore confirmed the presence of an RLFS in the *ORF16* promoter region of the KSHV genome. Interestingly, the viral RLFS tested were detected in both latently infected cells and cells undergoing lytic replication (Fig. 4B), suggesting that RLFS are present, albeit at different quantities, in both phases of the KSHV infectious cycle. Due to the repetitive nature of the TR (see Supplementary Table 5), qPCR was not suitable for measuring the specificity of R-loop precipitation in this region of the KSHV genome. However, traditional PCR clearly demonstrated the presence of TR sequences in samples immunoprecipitated with S9.6 antibody, while PCR amplification was not successful following precipitation with control Immunoglobulin G (IgG) (Fig. 4C). Moreover, treatment with RNase H resulted in the loss of S9.6 precipitation for TR sequences. Together, these results demonstrate that the RLFS prediction algorithm successfully identified bona fide RLFS in KSHV.

## Discussion

Computational prediction is a useful tool to identify possible R-loop and RNA-DNA hybrid forming loci. QmRLFS-finder is powerful in identifying known R-loops in many cellular organisms which are universally coded by dsDNA genomes^22,26^. In this study, for the first time, we use QmRLFS-finder to predict R-loop loci in the genomes of remarkably versatile viral families. To perform R-loop mapping and comprehensive analysis, only complete viral genomes were explored. Initially, we considered excluding genomes of RNA viruses that do not require DNA in their life cycle. However, the need for information about the structure and stability of R-loops has recently arisen with the development of antisense oligonucleotides as novel antiviral and anticancer therapeutics^39,40^. Therefore, we included viral RNA genomes in our study because we believed that information about RNA-DNA hybrid loci could be useful for designing probes/oligos to bind or avoid R-loop forming regions. Thus, all types of viral genomes were utilized for R-loop prediction in this study.

Here, apart from computational prediction, we also experimentally demonstrated the presence of R-loops in KSHV genomes. Since the function of RLFS in viral genomes is essentially undefined, with only a single experimental evaluation, this resource highlights a number of molecular elements that could be readily targeted using BAC mutagenesis to define their functions in regulating gene expression, genome organization, and ultimately pathogenesis. For instance, since the EBV origin of replication consists of RLFS, we hypothesize that the RLFS in the KSHV TR are similarly involved in replication of the latent viral episome. Interestingly, ChIP-seq for LANA binding sites revealed overlap with several predicted RLFS in the host genome (examples in Supplementary Fig. 6), suggesting that R-loops may serve as docking sites for LANA. Moreover, given the difficulty of defining R-loop function in cellular biology, it is worth noting that the compact nature of herpesvirus genomes and ease of mutagenesis makes these viruses useful tools for further defining R-loop functions in various cellular biology. This finding also opens an opportunity to study viral-host interaction via R-loop structure. Future observations that explore R-loop occurring *in vivo* may provide information on regulation of viral phases. The predicted RLFS in other viral genomes provide potential links to further understanding the mechanisms that regulate their infectious cycles.

While a previous study focused on the relationship between R-loop and genome instability of host cell^25^, this study showcases widespread R-loop formation in multiple viral genomes, including KSHV. R-loop formation during KSHV infection influences the host transcription DNA damage response (DDR) and genome instability^25^. The KSHV ORF57 protein hijacks the host transcription and export complex (hTREX) protein, an RNA binding protein that prevents R-loop formation. This induces DNA damage in KSHV infected cells^25^. We hypothesize that hijacking hTREX protein from host cells creates an environment for increasing R-loop formation in the KSHV genome.

While RLFSs are a common sequence feature in all members in *Herpesvirales*, it is interesting that no RLFS was predicted in any members of *Ligamenvirales*. *Ligamenvirales* are classified into two families, *Rudiviridae* and *Lipothrixviridae*. Both families consist of virus with linear dsDNA genomes. These viruses infect archaea found in extreme environments^41^. Previous studies have showed that all members in *Rudiviridae* have a non-G-rich motif, AATTTAGGAATTTAGGAATTT, as a common sequence feature at the termini of their linear genomes. This motif can form DNA hairpin structures which contribute to DNA replication^42^, precluding the need for R-loops for initial strand separation and loading of core replication proteins as seen in EBV^24^. No hairpin structures are found the termini of *Lipothrixviridae* genomes, however, the study of this virus family is still incipient and somewhat limited.

Results generated from our analyses drove us to explore the RLFSs in herpesvirus genomes, however, there are several other virus families that contain high ratio of RLFS-positive genomes. For example, *tymoviridae*, which cause severe agricultural losses in many parts of the world. There are no studies indicating R-loop formation in these viruses. However, there is an increasing number of reports on R-loop formation in plants, the natural host of *tymoviridae*^43,44^. RLFS data could be useful in guiding experimental efforts in global analysis of virus-host interactions in agriculturally important plants and their viral pathogens. This study presents a preliminary effort to explore the prevalence of R-loop in viral genomes. The generated RLFS prediction dataset can be used in a variety of dimensions, including in-depth investigation of the role of R-loops in host-pathogen interactions and manipulation of cellular processes by viruses.

## Supplementary information

Supplementary information.

Supplementary information.

## References

[1] Nordheim A, Rich A (1983). Negatively supercoiled simian virus 40 DNA contains Z-DNA segments within transcriptional enhancer sequences. Nature.

[2] McCarty DM, Young SM, Samulski RJ (2004). Integration of adeno-associated virus (AAV) and recombinant AAV vectors. Annu Rev Genet.

[3] Schnepp BC, Clark KR, Klemanski DL, Pacak CA, Johnson PR (2003). Genetic fate of recombinant adeno-associated virus vector genomes in muscle. J Virol.

[4] Metifiot M, Amrane S, Litvak S, Andreola ML (2014). G-quadruplexes in viruses: function and potential therapeutic applications. Nucleic Acids Res.

[5] Dethoff, E. A. *et al*. Pervasive tertiary structure in the dengue virus RNA genome. *Proc Natl Acad Sci USA*, 10.1073/pnas.1716689115 (2018). 10.1073/pnas.1716689115 PMC6233125 30341219

[6] Roy D, Lieber MR (2009). G clustering is important for the initiation of transcription-induced R-loops *in vitro*, whereas high G density without clustering is sufficient thereafter. Mol Cell Biol.

[7] Roy D, Yu K, Lieber MR (2008). Mechanism of R-loop formation at immunoglobulin class switch sequences. Mol Cell Biol.

[8] Chedin F (2016). Nascent Connections: R-Loops and Chromatin Patterning. Trends Genet.

[9] Graf M (2017). Telomere Length Determines TERRA and R-Loop Regulation through the Cell Cycle. Cell.

[10] Kuznetsov, V. A., Bondarenko, V., Wongsurawat, T., Yenamandra, S. P. & Jenjaroenpun, P. Toward predictive R-loop computational biology: genome-scale prediction of R-loops reveals their association with complex promoter structures, G-quadruplexes and transcriptionally active enhancers. *Nucleic Acids Res*, 10.1093/nar/gky690 (2018). 10.1093/nar/gky690 PMC6125685 30053183

[11] Wongsurawat T, Jenjaroenpun P, Kwoh CK, Kuznetsov V (2012). Quantitative model of R-loop forming structures reveals a novel level of RNA-DNA interactome complexity. Nucleic Acids Res.

[12] Ginno PA, Lott PL, Christensen HC, Korf I, Chedin F (2012). R-loop formation is a distinctive characteristic of unmethylated human CpG island promoters. Mol Cell.

[13] Powell WT (2013). R-loop formation at Snord116 mediates topotecan inhibition of Ube3a-antisense and allele-specific chromatin decondensation. Proc Natl Acad Sci USA.

[14] Yeo AJ (2014). R-Loops in Proliferating Cells but Not in the Brain: Implications for AOA2 and Other Autosomal Recessive Ataxias. PLoS One.

[15] Haeusler AR (2014). C9orf72 nucleotide repeat structures initiate molecular cascades of disease. Nature.

[16] Kannan A, Jiang X, He L, Ahmad S, Gangwani L (2020). ZPR1 prevents R-loop accumulation, upregulates SMN2 expression and rescues spinal muscular atrophy. Brain.

[17] Kannan A, Bhatia K, Branzei D, Gangwani L (2018). Combined deficiency of Senataxin and DNA-PKcs causes DNA damage accumulation and neurodegeneration in spinal muscular atrophy. Nucleic Acids Res.

[18] Perego MGL, Taiana M, Bresolin N, Comi GP, Corti S (2019). R-Loops in Motor Neuron Diseases. Mol Neurobiol.

[19] Yang Y (2014). Arginine methylation facilitates the recruitment of TOP3B to chromatin to prevent R loop accumulation. Mol Cell.

[20] Garcia-Muse T, Aguilera AR (2019). Loops: From Physiological to Pathological Roles. Cell.

[21] Ginno PA, Lim YW, Lott PL, Korf I, Chedin F (2013). GC skew at the 5′ and 3′ ends of human genes links R-loop formation to epigenetic regulation and transcription termination. Genome Res.

[22] Jenjaroenpun P, Wongsurawat T, Sutheeworapong S, Kuznetsov VA (2017). R-loopDB: a database for R-loop forming sequences (RLFS) and R-loops. Nucleic Acids Res.

[23] Sanz LA (2016). Prevalent, Dynamic, and Conserved R-Loop Structures Associate with Specific Epigenomic Signatures in Mammals. Mol Cell.

[24] Rennekamp AJ, Lieberman PM (2011). Initiation of Epstein-Barr virus lytic replication requires transcription and the formation of a stable RNA-DNA hybrid molecule at OriLyt. J Virol.

[25] Jackson BR, Noerenberg M, Whitehouse A (2014). A novel mechanism inducing genome instability in Kaposi’s sarcoma-associated herpesvirus infected cells. PLoS Pathog.

[26] Jenjaroenpun P, Wongsurawat T, Yenamandra SP, Kuznetsov VA (2015). QmRLFS-finder: a model, web server and stand-alone tool for prediction and analysis of R-loop forming sequences. Nucleic Acids Res.

[27] Zhang Q, Jun SR, Leuze M, Ussery D, Nookaew I (2017). Viral Phylogenomics Using an Alignment-Free Method: A Three-Step Approach to Determine Optimal Length of k-mer. Sci Rep.

[28] Federhen S (2012). The NCBI Taxonomy database. Nucleic Acids Res.

[29] Quinlan AR (2014). BEDTools: The Swiss-Army Tool for Genome Feature Analysis. Curr Protoc Bioinformatics.

[30] Brulois KF (2012). Construction and manipulation of a new Kaposi’s sarcoma-associated herpesvirus bacterial artificial chromosome clone. J Virol.

[31] Halasz L (2017). RNA-DNA hybrid (R-loop) immunoprecipitation mapping: an analytical workflow to evaluate inherent biases. Genome Res.

[32] Ye J (2012). Primer-BLAST: a tool to design target-specific primers for polymerase chain reaction. BMC Bioinformatics.

[33] Rigby RE (2014). RNA:DNA hybrids are a novel molecular pattern sensed by TLR9. EMBO J.

[34] Deng Z, Wang Z, Lieberman PM (2012). Telomeres and viruses: common themes of genome maintenance. Front Oncol.

[35] Yu K, Chedin F, Hsieh CL, Wilson TE, Lieber MR (2003). R-loops at immunoglobulin class switch regions in the chromosomes of stimulated B cells. Nat Immunol.

[36] Kaufer BB, Jarosinski KW, Osterrieder N (2011). Herpesvirus telomeric repeats facilitate genomic integration into host telomeres and mobilization of viral DNA during reactivation. J Exp Med.

[37] Arbuckle JH, Medveczky PG (2011). The molecular biology of human herpesvirus-6 latency and telomere integration. Microbes Infect.

[38] Toubiana S, Selig S (2018). DNA:RNA hybrids at telomeres - when it is better to be out of the (R) loop. FEBS J.

[39] Liang XH, Sun H, Nichols JG, Crooke ST (2017). RNase H1-Dependent Antisense Oligonucleotides Are Robustly Active in Directing RNA Cleavage in Both the Cytoplasm and the Nucleus. Mol Ther.

[40] Nakamori M, Gourdon G, Thornton CA (2011). Stabilization of expanded (CTG)*(CAG) repeats by antisense oligonucleotides. Mol Ther.

[41] Prangishvili D, Krupovic M (2012). A new proposed taxon for double-stranded DNA viruses, the order “Ligamenvirales”. Arch Virol.

[42] Vestergaard G (2005). A novel rudivirus, ARV1, of the hyperthermophilic archaeal genus Acidianus. Virology.

[43] Xu, W. *et al*. The R-loop is a common chromatin feature of the Arabidopsis genome. *Nat Plants*, 10.1038/s41477-017-0004-x (2017). 10.1038/s41477-017-0004-x 28848233

[44] Sun Q, Csorba T, Skourti-Stathaki K, Proudfoot NJ, Dean C (2013). R-loop stabilization represses antisense transcription at the Arabidopsis FLC locus. Science.

