# Author Correction: R-loop-forming Sequences Analysis in Thousands of Viral Genomes Identify A New Common Element in Herpesviruses

**DOI:** 10.1038/s41598-020-68537-7

**Published:** 2020-07-17

**Authors:** Thidathip Wongsurawat, Arundhati Gupta, Piroon Jenjaroenpun, Shana Owens, James Craig Forrest, Intawat Nookaew

**Affiliations:** 1 Department of Biomedical Informatics, College of Medicine, University of Arkansas for Medical Sciences, Little Rock, Arkansas USA; 2 Department of Microbiology and Immunology and Center for Microbial Pathogenesis and Host Inflammatory Responses, University of Arkansas for Medical Sciences, Little Rock, Arkansas USA; 3 Present Address: Department of Pediatrics, University of Pittsburgh School of Medicine, Pittsburgh, PA USA

Correction to: *Scientific Reports* 10.1038/s41598-020-63101-9, published online 14 April 2020

The original version of this Article contained a typographical error in the spelling of the author James Craig Forrest which was incorrectly given as J. Craig Forrest. This has now been corrected in the PDF and HTML versions of the Article, and in the accompanying Supplementary Information file.

