# Supplementary material for: R-loop-forming Sequences Analysis in Thousands of Viral Genomes Identify A New Common Element in Herpesviruses
Source: Sci Rep. 2020 Apr 14;10:6389. doi: 10.1038/s41598-020-63101-9 (PMC7156643; doi:10.1038/s41598-020-63101-9)
Supplement: Supplementary file 1 — Supplementary information. [file 41598_2020_63101_MOESM1_ESM.pdf]

Supplementary Figures : R-loop-forming Sequences Analysis in Thousands of Viral Genomes Identify A New Common Element in Herpesviruses.

Thidathip Wongsurawat [a,*], Arundhati Gupta [b,#], Piroon Jenjaroenpun [a], Shana Owens [b], J. Craig Forrest [b], Intawat Nookaew [a,*]

[a]Department of Biomedical Informatics, College of Medicine, University of Arkansas for Medical Sciences, Little Rock, Arkansas, USA

[b]Department of Microbiology and Immunology and Center for Microbial Pathogenesis and Host Inflammatory Responses, University of Arkansas for Medical Sciences, Little Rock, Arkansas, USA.

[#]Current address: Department of Pediatrics, University of Pittsburgh School of Medicine, Pittsburgh, PA, USA

* To whom correspondence should be addressed: Thidathip Wongsurawat; Email: twongsurawat@uams.edu and Intawat Nookaew; Email: inookaew@uams.edu

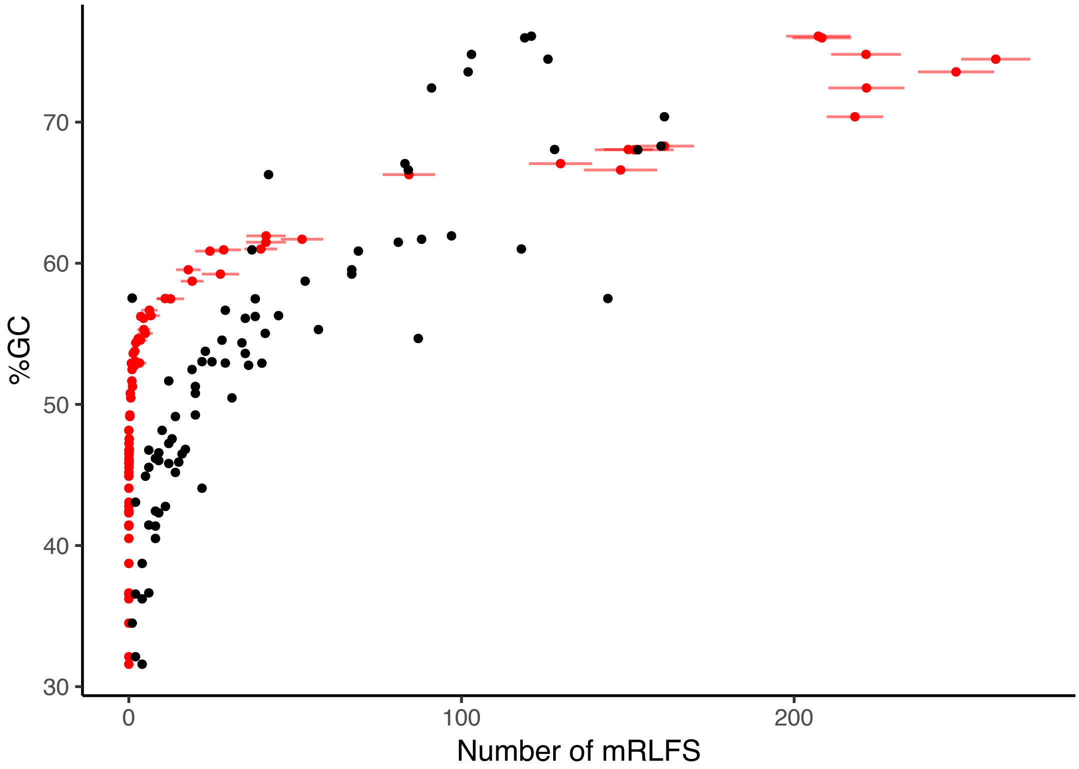

Supplementary Figure 1. Scatter plot show the relationship between %GC content and number of mRLFS of all 77 herpesvirus genomes in the reference dataset (black color). The red dots indicate the mean of mRLFS from the random shuffling of DNA sequences (n =30) from the individual herpesvirus by preserving GC content and genome length. Error bars depict standard error of the mean.

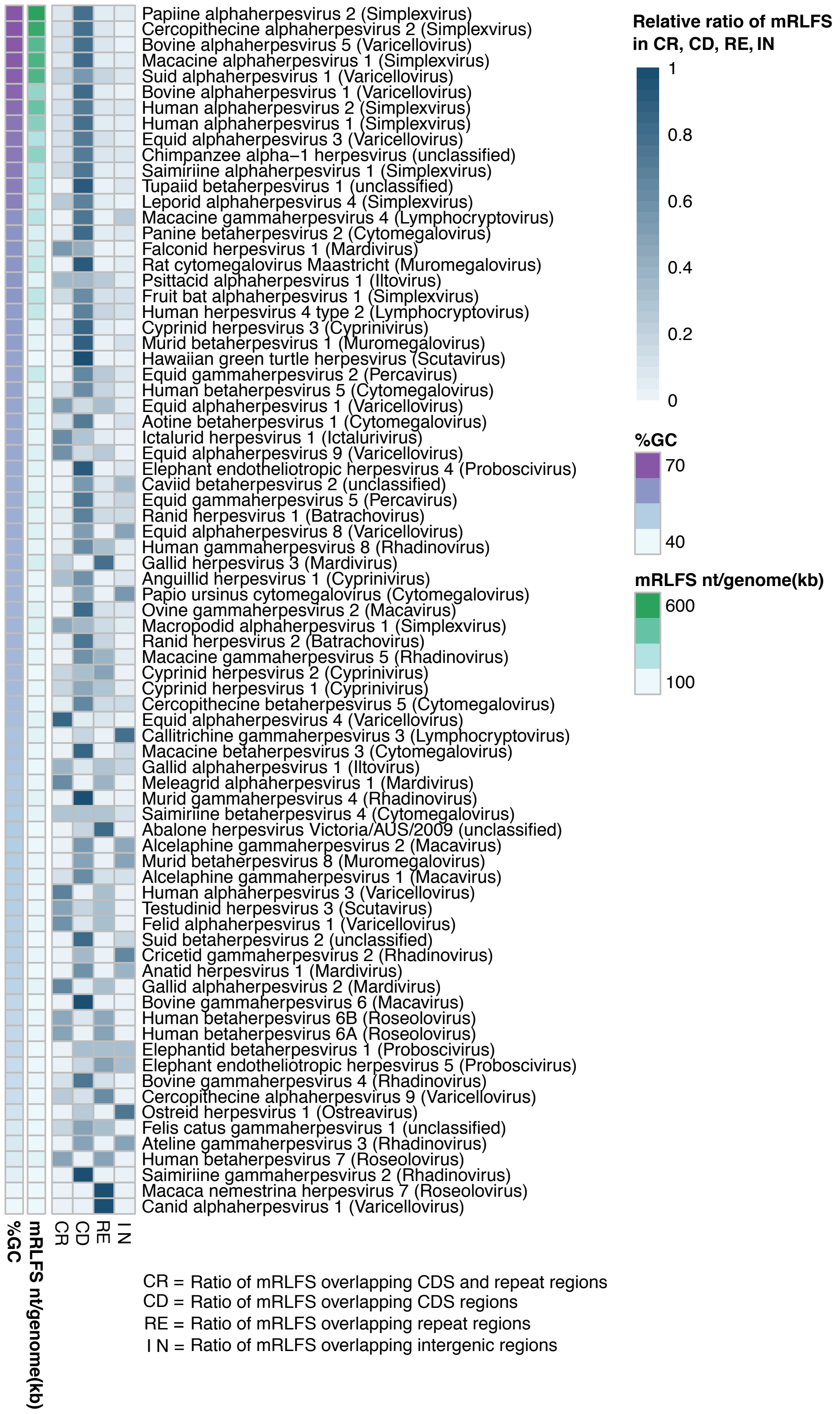

Supplementary Figure 2. RLFS number, coverage & ratio of RLFS in CDS and non-CDS in each herpesvirus genome.

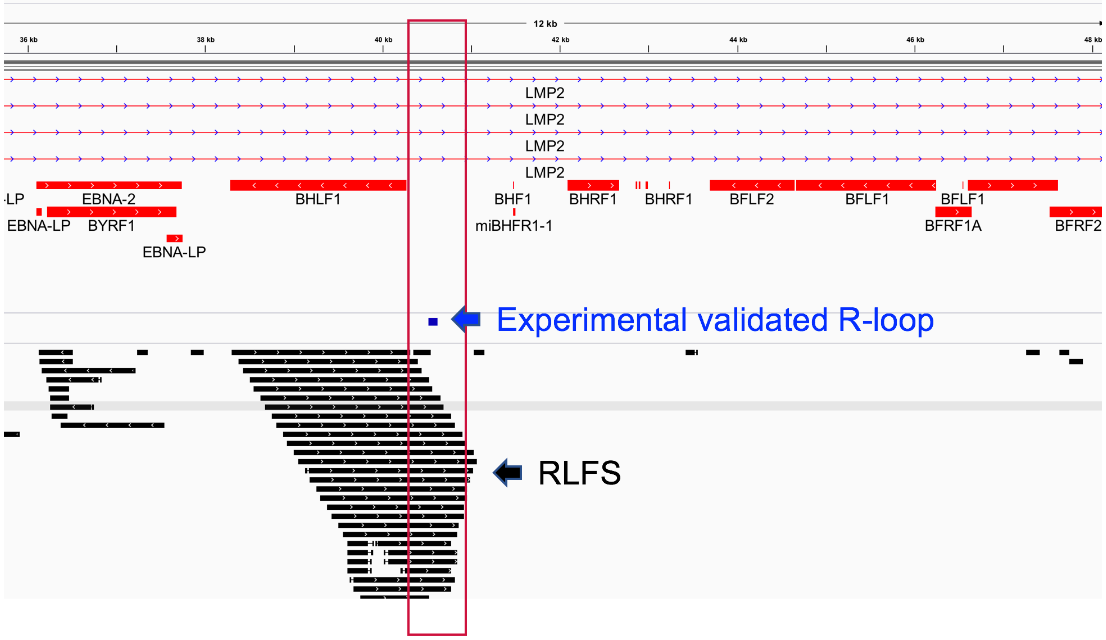

Supplementary Figure 3. Overlapping between RLFS and experimentally validated R-loop [20] was shown in red box.

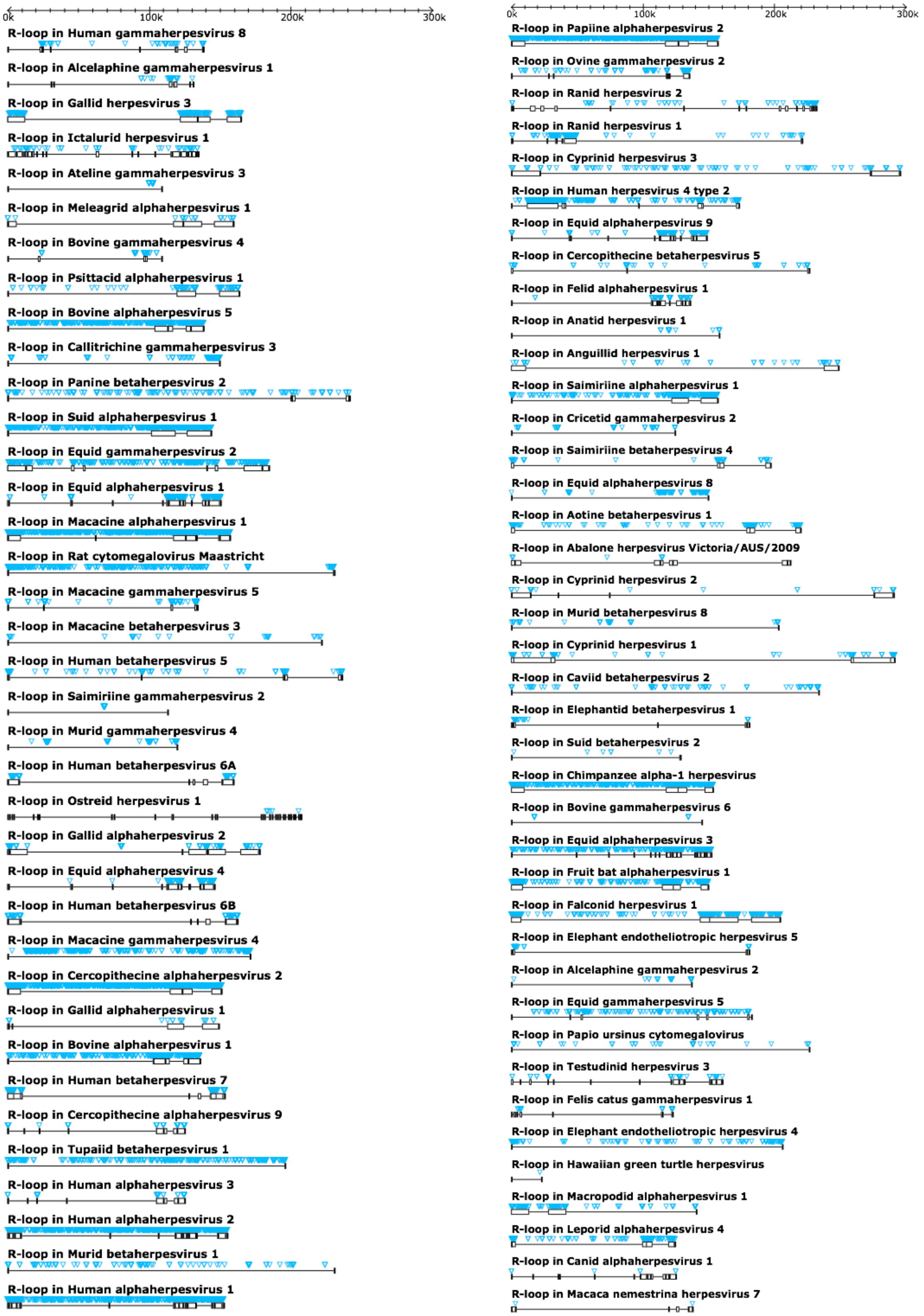

Supplementary Figure 4. Distribution of RLFS in 77 herpesvirus genomes. The black line represented the genomes. The blue triangle represented the genomic positions of RLFS. The open boxes represented the repeat regions in the genome.

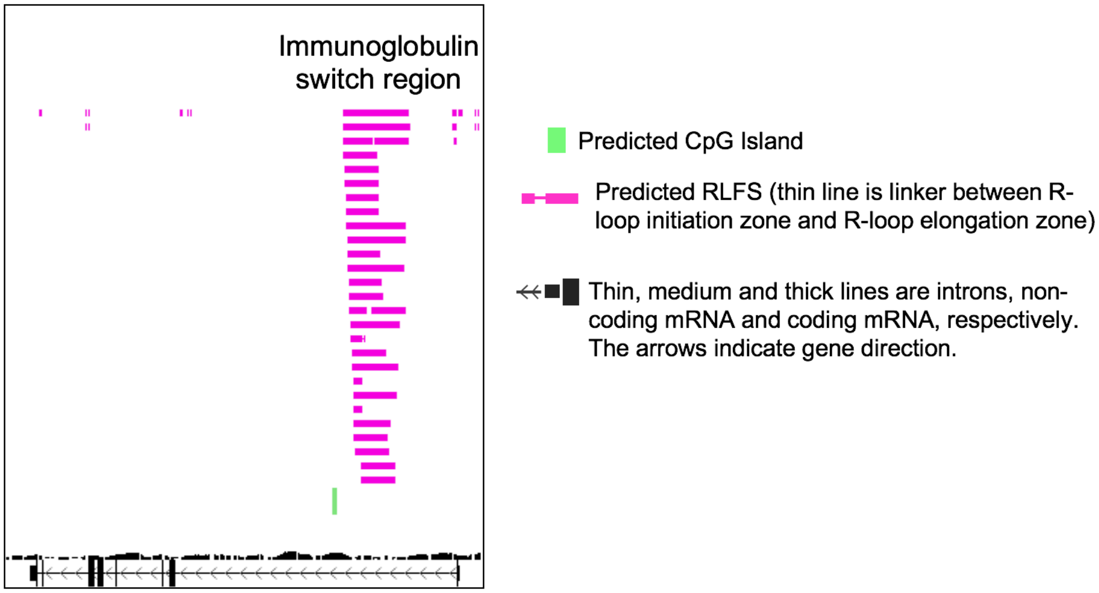

Supplementary Figure 5. Immunoglobulin switch region is an R-loop prone region

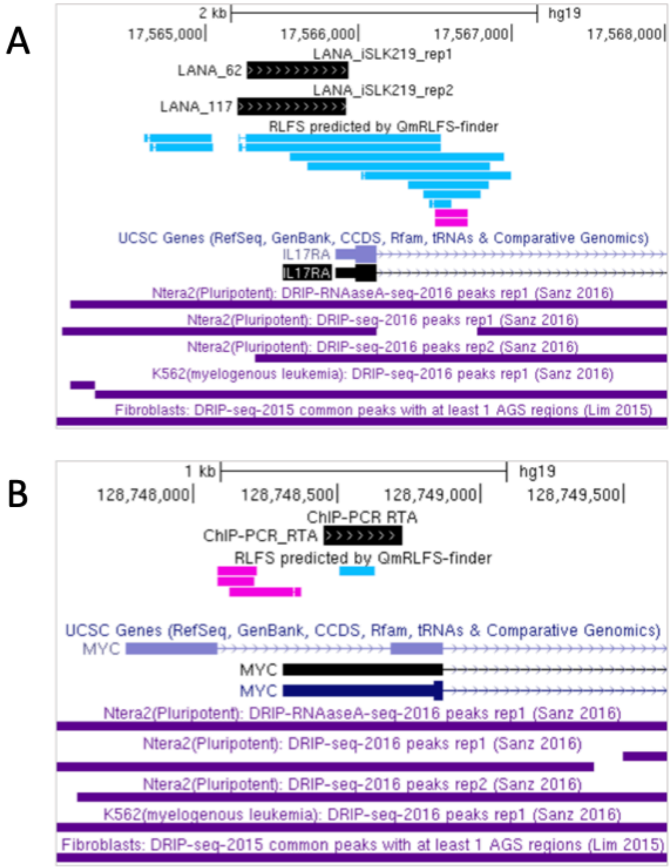

Supplementary Figure 6. Viral proteins target host-chromosome loci in the R-loop forming regions. Overlapping between RLFS, DNA-RNA immunoprecipitation - sequence (DRIP-seq) signal and known host-chromosome binding sites for viral LANA and Rta were observed. (A) KSHV-LANA binding site on promoters of IL-17 receptor (IL17RA) [32]. (B) EBV-Rta binding site on the regulatory regions of *MYC* [33].
